# Exploring motivational readiness for adapted physical activity in older adults with chronic illness: patterns of self-care behaviors, engagement, and psychological distress

**DOI:** 10.3389/fpsyg.2026.1796562

**Published:** 2026-03-11

**Authors:** Daniela Lemmo, Fabrizio Mezza, Alessandra Cuomo, Antonio Bianco, Antonella Di Donato, Vincenzo De Luca, Michele Virgolesi, Antonio Picone, Angela Palomba, Carlo Ruosi, Maddalena Illario, Guido Iaccarino, Maria Francesca Freda

**Affiliations:** 1 Department of Humanities, University of Naples Federico II, Naples, Italy; 2 Department of Clinical Medicine and Surgery, University of Naples Federico II, Naples, Italy; 3 Department of Public Health, University of Naples Federico II, Naples, Italy

**Keywords:** older adults, chronic illness, motivational readiness, health behavior change, clinical psychological intervention

## Abstract

**Introduction:**

In clinical settings, adapted physical activity (APA) is increasingly prescribed to older adults with chronic conditions, yet adherence remains low and motivational readiness varies widely at the time of prescription. While the Transtheoretical Model (TTM) describes stages of change for health behaviors, less is known about how motivational stage relates to broader psychological dimensions relevant to healthy aging, such as self-care behaviors, engagement in healthy aging promotion, and psychological distress.

**Methods:**

A cross-sectional study within the Age.it Project was conducted with 74 older adults (mean age ≈ 70.82 years) attending outpatient clinics at the University Hospital Federico II and prescribed APA. Motivational stage was assessed through the MAC2-R AF. Self-care behaviors were measured with the Self-Care Inventory; Engagement in healthy aging promotion with the EHAP-S; Psychological distress with the K10. Group differences across motivational stages were tested using Kruskal–Wallis analyses with Dunn–Bonferroni post-hoc comparisons and epsilon squared effect sizes.

**Results:**

Most participants were classified in Contemplation (59.5%), followed by Determination (23%) and Precontemplation (17.6%). No participants were in Action or Maintenance stages. Motivational stage was significantly associated with self-care monitoring, engagement, and distress, with large effects. Contemplation showed higher self-care monitoring and higher distress, alongside lower engagement. Engagement was higher in Precontemplation and Determination compared to Contemplation, whereas distress increased progressively from Precontemplation to Determination.

**Discussion:**

Findings suggest distinct, non-linear psychological configurations within the motivational stages at the time of APA prescription. Contemplation emerges as a vulnerable phase characterized by symptom surveillance and emotional burden without engagement in healthy aging promotion; Determination combines higher engagement with heightened distress; Precontemplation may reflect stable self-care routines and low distress but potential resistance to integrating exercise into self-care identity. Integrating motivational stage assessment with psychological–clinical indicators may support more personalized, motivated, and sustainable APA prescriptions for older adults.

## 1 Introduction

Population aging represents one of the major challenges for contemporary healthcare systems. Increased life expectancy is accompanied by a growing prevalence of chronic diseases, multimorbidity, and functional limitations, which significantly impact the quality of life, autonomy, and psychological well-being of older people (World Health Organization, 2020; Beard et al., 2016). In this scenario, physical inactivity is recognized as one of the main modifiable risk factors, associated with worse health outcomes, greater disability and increased use of health services (Guthold et al., 2018; Piercy et al., 2018). In recent decades, adapted physical activity (APA) and exercise prescription have been progressively integrated into the prevention and management of chronic diseases, becoming key interventions for supporting healthy aging (Di Mare et al., 2025; Eckstrom et al., 2020; de Mello et al., 2019). In addition to the widely documented physiological benefits, physical activity interventions in older age are increasingly conceptualized as complex behavioral prescriptions, requiring sustained motivation, psychological engagement, and self-regulation skills (Phillips et al., 2020; Franco et al., 2015). However, adherence to physical activity among older adults remains low overall, especially when exercise is prescribed in a clinical setting rather than chosen as a recreational or independent activity (Schutzer and Graves, 2004; Picorelli et al., 2014). From a clinical health psychology perspective, understanding the older adults’ motivational readiness to health behavioral change when physical activity is promoted or prescribed is crucial for designing effective and sustainable interventions over time.

Motivation to change lifestyle and health behaviors is a dynamic and multidimensional process, particularly complex in late life, when experiences of illness, health beliefs, established habits and perceptions of vulnerability interact with each other (Rhodes et al., 2017).

The Transtheoretical Model of Change (TTM, Prochaska et al., 1992, 2005) has been widely used to study the adoption and maintenance of physical activity in older adults. According to this model, individuals progress through qualitatively distinct motivational stages—precontemplation, contemplation, determination (or preparation), action, and maintenance—each characterized by specific cognitive, emotional, and behavioral patterns.

Precontemplation is characterized by unawareness, denial, low perceived relevance, and lack of motivation to change. Contemplation involves problem awareness, ambivalence, decisional conflict, and heightened emotional load, without behavioral commitment. During Determination stage, individuals show increased motivation, intentionality, and cognitive engagement, alongside planning efforts. The Action stage is marked by active behavioral implementation, increased self-efficacy, and goal-directed regulation. Finally, Maintenance reflects behavioral consolidation, adaptive self-regulation, and relapse prevention (Raihan and Cogburn, 2023).

Numerous studies indicate that a significant proportion of older adults who have been prescribed physical activity are in the early or intermediate motivational stages, particularly precontemplation and contemplation (Marcus et al., 2000). These stages are often characterized by ambivalence, low confidence in one’s ability to change, and growing awareness of the discrepancy between current behaviors and health goals (Pietrabissa et al., 2012, 2013, 2015). The changes in physical activity in later life is shaped by a balance between motivational facilitators and perceived barriers. Facilitators include an active self-identity, perceived physical and emotional benefits, social support, access to safe environments, and personalized programs that enhance self-efficacy (O’Neil-Pirozzi et al., 2022; Collado-Mateo et al., 2021). Conversely, barriers such as fear of falling, chronic pain, low confidence, social isolation, and unfavorable environments may hinder engagement. Additionally, age-related stereotypes and internalized ageism can further reduce openness to physical activity in older adults (Huffman and Amireault, 2021; Donizzetti and Capone, 2023; Donizzetti et al., 2024). It is important to emphasize that the prescription of physical activity does not necessarily equate to a real willingness to change, making it essential to assess the motivational phase to avoid standardized and ineffective approaches (Hawley-Hague et al., 2016).

Despite the widespread use of stage models in physical activity research, limited attention is still paid to the relationship between motivational stage and broader psychological dimensions of aging, such as self-care practices, engagement in promoting healthy aging, and psychological distress—factors that may critically influence the effectiveness of exercise prescriptions in clinical settings. According to the Middle-Range Theory of Self-Care, self-care comprises a set of learned behaviors aimed at maintaining health (self-care maintenance), monitoring symptoms (self-care monitoring), and managing disease-related changes (self-care management) (Riegel et al., 2012; 2018). From this perspective, physical activity—especially when prescribed as part of treatment pathways—requires continuity in self-care maintenance (e.g., routine adherence to exercise) and self-care monitoring (e.g., observation of bodily responses, fatigue, and symptomatic changes).

Previous studies have shown that greater self-care skills are associated with better functional outcomes, reduced symptom burden, and improved psychological adjustment in chronic conditions (Buck et al., 2015; Luciani et al., 2022). However, engaging in physical activity as a self-care practice can be psychologically demanding, particularly in the initial stages of change, when individuals are still negotiating the meaning, feasibility, and personal relevance of exercise in their daily lives. Analyzing how self-care behaviors vary according to the motivational phase can therefore offer relevant insights into the psychological processes underlying adherence to adapted physical activity programs in older adults. Alongside self-care, the concept of engagement in healthy aging promotion emphasizes the active and intentional involvement of individuals in maintaining their physical, psychological, and social well-being over time (Menichetti et al., 2016, 2018). Engagement reflects not only behavioral activation, but also a sense of agency, purpose, and subjective investment in one’s aging process. Higher levels of engagement in healthy aging have been associated with healthier lifestyles, improved emotional regulation, and a greater sense of control in older adults (Barello et al., 2020). At the same time, behavioral changes in old age can be accompanied by increased psychological distress. Confronting chronic conditions, functional decline, and medical prescriptions can trigger emotional responses such as anxiety, frustration, or demoralization. Distress can act both as a barrier and as a potential driver of change, depending on its intensity and available coping resources (Kessler et al., 2002). Despite the growing recognition of the emotional dimension in lifestyle change processes, few studies have jointly examined motivational status, self-care, engagement in healthy aging, and psychological distress in older adults prescribed adapted physical activity. An integrated psychological perspective is therefore needed to grasp the complexity of these processes.

This study aims to provide a preliminary and cross-sectional snapshot of motivational readiness for physical activity in a sample of older adults at the specific clinical moment in which adapted physical activity is prescribed. In particular, by adopting the motivational stages derived from the Transtheoretical Model of behavior change as a descriptive lens, this study explores psychological correlates at the time of prescription, examining how each motivational positioning may be associated with self-care behaviors, engagement in healthy aging promotion, and psychological distress in this specific clinical context.

Specifically, the study aims to: (a) Analyze how people over 65 with chronic diseases are distributed in the different motivational stages following a medical prescription of adapted physical activity; (b) Explore how levels of self-care, engagement and distress vary along these motivational stages for readiness to change.

Based on theoretical models of health behavior change, as and self-care, as well as available empirical evidence, the following hypotheses have been formulated:

1. Self-care behaviors will differ according to the motivational stage, with higher levels of self-care maintenance and monitoring among individuals in the more advanced stages (Determination) compared to those in the Precontemplation and Contemplation stages.
2. Engagement in healthy ageing promotion will vary according to the motivational stage, reflecting different levels of proactive involvement in health behaviors.
3. Psychological distress will be higher in the intermediate motivational stages (Contemplation and Determination), where awareness of the need for change and perceived demands are greater, compared to the Precontemplation stage.

Given the exploratory and clinic-based nature of the study, findings are not intended to generalize to the broader population of older adults with chronic illness, nor to validate the Transtheoretical Model as a full developmental trajectory. Rather, they provide a contextualized cross-sectional snapshot of psychological configurations associated with motivational positioning at the time of medical prescription of APA.

## 2 Materials and methods

### 2.1 Study design and setting

This cross-sectional study was conducted between May 2024 and February 2025 at the Physical Medicine and Sports Medicine outpatient clinics of the University Hospital Federico II (Naples, Italy). Participants were older adults (≥60 years) who had been prescribed physical activity for at least 3 months. Participants were assessed at the time of initial prescription of adapted physical activity during their outpatient visit. The evaluation therefore captured motivational readiness prior to the initiation of regular physical activity practice, rather than after behavioral implementation. Eligible participants were Italian residents able to understand and complete the questionnaires independently and to provide written informed consent. Exclusion criteria included a clinical diagnosis of dementia or severe psychiatric disorders, based on information available in patients’ medical records and on the clinical judgment of the treating physicians within the outpatient setting.

### 2.2 Measures

*Socio-demographic* were collected, including age, gender, education, marital status, presence of chronic illness. Education comprised the following categories: no formal education; primary school; lower-secondary school; vocational qualification; bachelor’s degree; master’s degree; and doctorate. Marital status comprised the following categories: single; cohabiting; separated or divorced; married; and widowed.

*Health variables* comprised presence of a chronic illness (yes/no) and self-rated health, assessed using a single-item question asking participants to evaluate their overall health on a 5-point Likert-type scale (1 = *Very bad*, 2 = *Bad*, 3 = *Medium*, 4 = *Good*, 5 = *Very good*). Higher scores indicate better perceived health status. This single-item self-assessment has been widely used as an indicator of general health perception and has shown good validity as a global indicator of health status (Cislaghi and Cislaghi, 2019).

*Motivational readiness for physical activity* was assessed using the 18-item MAC2-R AF questionnaire (Spiller et al., 2009), developed and validated in Italy to assess motivation for change based on the Transtheoretical Model (Prochaska and Velicer, 1997). The questionnaire captures three core components of motivation based on the transtheoretical model: Readiness to Change, Discrepancy, and Self-efficacy. Each item is rated on a 7-point Likert scale ranging from 0 (*Not at all true*) to 6 (*Completely true*). The instrument demonstrated acceptable to excellent internal consistency for its subscales in the original validation study (Cronbach’s α = 0.61–0.87). For the purposes of this study, we focused solely on the component of Readiness to Change, which includes five motivational stages: Precontemplation, Contemplation, Determination, Action, and Maintenance. Each participant was assigned to a dominant stage based on their response profile.

*Engagement in healthy aging promotion* was measured using the 8-item Engagement in Healthy Aging Promotion Scale (EHAP-S; Menichetti et al., 2018). Items were answered on a 5-point Likert scale from 1 (*strongly disagree*) to 5 (*strongly agree*). The total score is the sum of item responses (range: 8–40), with higher scores indicating greater engagement in healthy aging promotion. In the present study, the scale showed excellent internal consistency (Cronbach’s α = 0.95).

*Self-care behaviors* was assessed using the Self-Care Inventory (SCI; Riegel et al., 2018), developed to measure self-care behaviors according to the Middle Range Theory of Self-Care (Riegel et al., 2012). The SCI consists of three subscales assessing different dimensions of self-care: Self-Care Maintenance (seven items), Self-Care Monitoring (six items), and Self-Care Management (six items). Each item is rated on a 5-point Likert scale ranging from 1 (*Never or rarely*) to 5 (*Always or daily*). The instrument was adapted and psychometrically evaluated in the Italian population (Luciani et al., 2022), showing good validity and internal consistency. For the purposes of the present study, only the Self-Care Maintenance and Self-Care Monitoring subscales were used. Internal consistency for each subscale in the present sample was excellent for the Self-Care Maintenance (α = 0.97) and Self-Care Monitoring (α = 0.93) subscales.

*Psychological distress* over the past 30 days was assessed using the 10-item Kessler Psychological Distress Scale (K10; Kessler et al., 2002). Each item captures symptom frequency on a 5-point Likert scale ranging from 1 (*None of the time*) to 5 (*All of the time*). The total score is the sum of item scores (range: 10–50), with higher scores indicating greater distress. We employed the validated Italian adaptation (Carrà et al., 2011), which has demonstrated robust psychometric properties. Internal consistency in the present sample was good (Cronbach’s α = 0.88).

### 2.3 Data analyses

Statistical analyses were performed using the IBM Statistical Package for Social Science (SPSS), version 30.0. The assumptions of normality and homogeneity of variances were assessed using the Shapiro–Wilk and Levene’s tests, respectively. Due to the violation of both assumptions, non-parametric tests were performed. Descriptive analysis of categorical variables is presented in total number and percentages and continuous variables as mean (SD) or median [interquartile range (IQR)], as appropriate for their distribution.

Kruskall-Wallis was performed to examine group differences across motivational stages, with Self-care Maintenance, Self-Care Monitoring, Engagement in Healthy Aging Promotion and Psychological Distress, as dependent variable.

*Post-hoc* pairwise comparisons were subsequently conducted using Dunn’s test with Bonferroni adjustment applied to adjust for multiple comparisons. Statistical significance was set at *p* < 0.05 (two-tailed) for all analyses. To evaluate the strength of the observed differences, effect sizes were calculated using epsilon squared (ε^2^). Statistical significance was set at *p* < 0.05 (two-tailed) for all analyses.

## 3 Results

### 3.1 Sample characteristics

The final analytic sample consisted of 74 older adults (*M* = 70.82 years, SD = 6.11). Of these, 68.9% were men (*n* = 51) and 31.1% were women (*n* = 23).

Regarding educational attainment, 23% of participants reported no formal education, 13.5% had completed middle school, 9.5% primary school, 10.8% held a vocational qualification, and 43.3% had a university degree (17.6% bachelor’s, 10.8% master’s, and 14.9% doctorate). With respect to marital status, 28.4% of the sample were single, 8.1% cohabiting, 23% separated or divorced, 14.9% married, and 25.7% widowed. In terms of occupational status, 35.1% were employed, 36.5% retired, and 28.4% unemployed. Self-rated general health varied across the sample: 13.5% described their health as excellent, 28.4% as very good, 17.6% as good, 16.2% as fair, and 24.3% as poor.

These distributions likely reflect the specific clinical recruitment context, in which participants were referred to a specialized outpatient service for adapted physical activity, and may therefore not be fully representative of the broader population of older adults with chronic illness.

Regarding motivational stages, the majority of participants (59.5%) of the sample was classified in the Contemplation stage (*n* = 44), followed by 23% in the Determination stage (*n* = 17) and 17.6% in the Pre-contemplation stage (*n* = 13). No participants were classified in the Action or Maintenance stages, consistent with the fact that assessment took place at the time of prescription of adapted physical activity, prior to behavioral initiation or consolidation.

Self-care maintenance mean scores were below the recommended threshold of 70 (*M* = 54.01, SD = 33.33; Riegel et al., 2009), whereas Self-care monitoring scores were higher (*M* = 75.95, SD = 27.14). Mean scores for engagement in healthy aging promotion were moderate (*M* = 22.53, SD = 10.40). Psychological distress levels were also in the moderate range (*M* = 33.31, SD = 9.05). Descriptive statistics for the main variables are presented in Table 1.

**Table 1:** Descriptive statistics of study variables for the study sample (*n* = 74).

Variables	Statistics
Age, M (SD; range)	69.05 (7.85; 50–92)
Sex, *n* (%)	
Women	23 (31.1%)
Men	51 (68.9%)
Education, *n* (%)	
No education	17 (23%)
Primary education	7 (9.5%)
Lower secondary education	10 (13.5%)
Vocational qualification	8 (10.8%)
Upper secondary education	13 (17.6%)
Bachelor’s degree	8 (10.8%)
Master’s degree	11 (14.9%)
Postgraduate qualification	
Marital status, *n* (%)	
Married or civil partnership	11 (14.9%)
Widowed	19 (25.7%)
Co-habiting	6 (8.1%)
Single	21 (28.4%)
Divorced/separated	17 (23.0%)
Presence of chronic illness (hypertension and/or osteoarthritis), *n* (%)	
No	18 (24.3%)
Yes	56 (75.7%)
Self-rated health, *n* (%)	
Excellent	10 (13.5%)
Very Good	21 (28.4%)
Good	13 (17.6%)
Fair	12 (16.2%)
Poor	18 (24.3%)
Motivational stage, *n* (%)	
Precontemplation	13 (17.6%)
Contemplation	44 (59.5%)
Determination	17 (23%)
Self-care maintenance, M (SD)	54.00 (33.33)
Self-care monitoring, M (SD)	75.94 (27.14)
Engagement in healthy aging promotion, M (SD)	22.52 (10.40)
Psychological distress, M (SD)	33.31 (9.05)

### 3.2 Comparisons of self-care maintenance, self-care monitoring, engagement in healthy aging promotion, and psychological distress across motivational stages

For self-care monitoring, participants in the Contemplation stage scored significantly higher than both Precontemplation and Determination (both *p* < 0.001), while no significant difference emerged between Precontemplation and Determination (*p* = 0.309). A similar pattern was observed for engagement in healthy aging promotion, with Precontemplation and Determination reporting significantly higher scores than Contemplation (both *p* < 0.001). No significant difference was observed between Precontemplation and Determination (*p* = 0.946). Finally, psychological distress increased progressively across motivational stages: participants in Precontemplation reported significantly lower distress than those in Contemplation and Determination (both *p* < 0.001), and distress levels were also higher in Determination compared to Contemplation (*p* = 0.025). Overall, motivational stage accounted for a large proportion of variance across outcomes, with ε^2^ ranging from 0.52 to 0.69. Median values (Mdn, IQR), Kruskal–Wallis statistics (H) and effect sizes (ε^2^) are presented in Table 2. Dunn–Bonferroni adjusted *p*-values for pairwise comparisons across motivational stages are presented in Table 3.

**Table 2:** Kruskal–Wallis H statistics, ε^2^ effect sizes for self-care maintenance, self-care monitoring, engagement in healthy aging promotion, and psychological distress across motivational stages.

Variable/group	Mdn (IQR)	Omnibus test		
		H	ε^2^	*p*-value
Self-care maintenance		50.75	0.69	<0.001
Precontemplation	100.00 (3.57)			
Contemplation	26.78 (14.29)			
Determination	85.71 (16.07)			
Self-care monitoring		48.5	0.67	<0.001
Precontemplation	30.00 (10.00)			
Contemplation	95.00 (10.00)			
Determination	65.00 (50.00)			
Engagement in healthy aging promotion		51.58	0.69	<0.001
Precontemplation	36.00 (2.00)			
Contemplation	14.00 (3.75)			
Determination	33.00 (4.50)			
Psychological distress		38.65	0.52	<0.001
Precontemplation	17.00 (3.50)			
Contemplation	36.00 (8.00)			
Determination	40.00 (5.00)			

Mdn, median; IQR, interquartile range; H, Kruskal–Wallis H statistic; ε^2^, epsilon squared (effect size); *p*-value, two-tailed significance value.

**Table 3:** Dunn–Bonferroni pairwise comparisons for self-care maintenance, self-care monitoring, engagement in healthy aging promotion, and psychological distress across motivational stages.

Variable/group	*Post hoc* tests		
	Contrast	Dunn test	*p*-value
Self-care maintenance	Contemplation – Determination	−4.700	0.000
	Contemplation – Precontemplation	6.366	0.000
	Determination – Precontemplation	1.811	0.210
Self-care monitoring	Precontemplation – Determination	−1.631	0.309
	Precontemplation – Contemplation	−6.157	0.000
	Determination – Contemplation	4.702	0.000
Engagement in healthy aging promotion	Contemplation – Determination	−5.335	0.000
	Contemplation – Precontemplation	5.998	0.000
	Determination – Precontemplation	1.004	0.946
Psychological distress	Precontemplation – Contemplation	−4.783	0.000
	Precontemplation – Determination	−6.143	0.000
	Contemplation – Determination	−2.638	0.025

*Post hoc* tests = Dunn’s multiple-comparisons test with Bonferroni correction (adjusted *p*-values).

## 4 Discussion

This study explored how self-care behaviors, engagement in healthy aging promotion, and psychological distress are distributed across the different motivational stages of physical activity change in older adults with chronic conditions who had been prescribed adapted physical activity. In the context of this preliminary study, the motivational stages were used as descriptive lenses to capture older adults’ motivational and psychological differences at a specific clinical entry point.

The distribution of participants shows a prevalence of the Contemplation stage (59.5%), followed by Determination (23%) and Precontemplation (17.6%).

First, the absence of participants classified in the Action and Maintenance stages should be interpreted in light of the specific clinical context and timing of data collection. Participants were assessed at the moment in which adapted physical activity was newly prescribed, meaning that behavioral change had just been proposed rather than already initiated or consolidated. Within this context, the absence of Action and Maintenance stages reflects early motivational positioning at the entry point of a clinically prescribed change process, rather than an indication of classification failure or low adherence. Importantly, the present findings should therefore be interpreted within the “pre-action” segment of the Transtheoretical Model. The observed psychological patterns primarily inform motivational processes operating in Precontemplation, Contemplation, and Determination stages, rather than the full trajectory toward sustained action and maintenance. In this sense, the study contributes to understanding early-stage motivational dynamics in a clinical prescription context, where readiness assessment may be particularly relevant for personalized interventions.

This concentration in the pre-action stages is consistent with the literature describing, in the elderly, a large proportion of people placed in the initial stages of change with respect to physical activity, and confirms the usefulness of stage models (TTM) to assess the readiness for health behavior in this population (Jiménez-Zazo et al., 2020). However, the joint analysis of psychological and behavioral variables highlights non-linear configurations, which suggest a more complex reading of the motivational process.

The Contemplation phase emerges as the most numerous and simultaneously as one of the most critical. Participants in this phase show significantly higher levels of self-care monitoring and psychological distress, associated with a low level of engagement in healthy aging promotion. This profile is particularly interesting because it suggests that awareness of the problem and attention to bodily signals do not automatically translate into active involvement in change. From a theoretical standpoint, contemplation is a reflective and ambivalent phase: the person recognizes the need to change behavior but has not yet translated this awareness into action. In this context, low engagement can be interpreted as an discrepancy between “thinking about change” and “feeling involved in change.” In this sense, low engagement in healthy aging may align with the very nature of contemplation: attention is focused on the problem (and its consequences), but the active investment in change remains limited. The fact that this phase is characterized by high symptom monitoring and significant distress suggests that the focus is predominantly directed at the problem and its possible consequences, rather than the development of active and agentic strategies for change.

A second, clinically relevant level of interpretation regarding the co-occurrence of monitoring and distress considers Contemplation as the stage in which chronic conditions are experienced with greater emotional burden and hyper-focus on bodily signals (“symptom surveillance”), without translating into a sense of direction or mastery. In other words, monitoring may take on a more “reactive” than self-regulatory quality: high attention to the body, but not yet organized into effective change strategies.

This configuration aligns with the literature on the “intention–behavior gap”: in pre-action stages, even when intention or awareness increases, translation into behavior may remain blocked if planning resources, self-efficacy, and emotional support are lacking (Massie et al., 2022). In this framework, engagement can function as a “motivational-agent” lever: if it is low, the individual may remain trapped in prolonged contemplation, where the discrepancy between “I know I should” and “I can’t do it” grows. In this perspective, contemplation can represent the most vulnerable phase of the change process, where “thinking about change without acting,” especially in the presence of distress and hyper-monitoring, can increase the risk of motivational stagnation.

The Determination phase shows a different but equally complex profile: in this stage, high levels of engagement in healthy aging emerge, but they are accompanied by significant psychological distress, even higher than that observed in contemplation. Traditionally, determination is associated with greater decision-making clarity and reduced distress; however, the results of this study suggest a possible paradoxical effect. One useful interpretation could be that of “anticipatory” distress or performance-related anxiety: when a person moves from “thinking” to “having to do,” the fear of failure, of not being capable, or of worsening their condition may arise. This is particularly true in advanced age and in the presence of chronic conditions, where movement can be associated with risk, pain, or instability. A key example is the fear of falling, which is frequently reported in older adults and is associated with anxiety and avoidance of physical activity due to fear of losing autonomy (Birhanie et al., 2021; Britting et al., 2024). In this sense, increased engagement may coexist with increased distress, fueled by performance anxiety, fear of failure, or concerns about movement (pain, falls, fatigue). The Determination phase could therefore represent an emotionally intense stage, where involvement grows but is not yet adequately supported by confidence in one’s abilities and emotional regulation strategies.

A particularly interesting finding concerns the Precontemplation phase, characterized by high levels of self-care maintenance and engagement in healthy aging, associated with low levels of psychological distress. This profile suggests that some older adults may perceive their care routines as effective and sufficient, not recognizing physical activity as a necessary, priority change or consistent with their self-care approach. In other words, this phase highlights that one can be “engaged” and active in promoting health according to their usual repertoire (medications, check-ups, diet, daily strategies), but not consider physical exercise change as part of their self-care. From this perspective, well-established self-care practices and a good level of engagement in health promotion can paradoxically transform into a resistance factor to change, when the prescription of physical activity is perceived as unnecessary or incongruent with the person’s identity and habitual strategies (Oyserman et al., 2007; Verplanken and Orbell, 2003). This interpretation is consistent with the idea that health behaviors are organized into systems of habits and beliefs: some established practices may facilitate adherence but can also act as “anchors” that reduce openness to new prescriptions if these are not perceived as coherent or useful.

Taken together, the results indicate that motivation for physical activity change does not evolve linearly across motivational phases. Self-care, engagement, and distress assume different configurations depending on the stage, highlighting how simple motivational advancement does not automatically guarantee increased active involvement or reduced emotional burden. These findings further suggest that different motivational stages are associated with distinct psychological configurations rather than with a simple gradient of improvement or deterioration. In this cross-sectional snapshot, higher distress in certain stages may reflect increased cognitive and emotional activation related to health concerns, perceived risks, and responsibility toward change. From this perspective, distress does not necessarily indicate maladaptive functioning, but may represent an emotionally salient positioning within the motivational process.

In particular, contemplation emerges as a fragile phase, determination as an emotionally dense stage, and precontemplation as a phase of stability potentially resistant to change. This “configuration-based” interpretation aligns with the literature, which highlights how TTM stages describe motivational positions, but do not automatically guarantee behavioral activation, especially in the presence of emotional barriers and health- and physical-functioning-related fears (Jiménez-Zazo et al., 2020).

### 4.1 Limitations

This study has some limitations that must be considered in the interpretation of the results. First, the cross-sectional design does not allow for causal conclusions or the observation of actual transitions between motivational phases over time. Second, the sample size and the concentration of participants in pre-action phases limit the ability to explore action and maintenance stages in depth. Third, the sample was recruited in a specific clinical context, which may reduce the generalizability of the results to other settings or non-clinical elderly populations. The study was conducted in an outpatient clinic and relied on a convenience, clinic-based sample. As participants were referred to a specialized service for adapted physical activity, selection bias cannot be excluded. The sample may differ from community-dwelling older adults with chronic illness in terms of health awareness, self-care behavior, or motivational orientation. In addition, the observed sex distribution and educational heterogeneity may reflect specific socio-cultural and referral patterns within the local healthcare context, limiting generalizability to other settings or populations. Future multi-center and community-based studies are needed to enhance external validity and examine potential socio-cultural variations in motivational readiness.

Fourth, health status was assessed only through the presence of chronic illness (yes/no) and a single-item measure of self-rated health, without detailed information on the type and severity of chronic conditions, thus limiting the clinical characterization of the sample. Finally, no formal cognitive or psychiatric screening was conducted, and the exclusion of participants with dementia or severe psychiatric disorders relied on clinical documentation and the judgment of treating physicians within the outpatient setting. This may have introduced some uncertainty regarding the accuracy of self-reported measures, underscoring the need for brief standardized assessments in future studies.

Despite these limitations, the study provides relevant insights into the psychological processes associated with the prescription of adapted physical activity in older adults.

### 4.2 Implications for clinical practice

The results of this study suggest that the assessment of the motivational stage should be accompanied by an analysis of the psychological and emotional resources associated with each stage. In particular, Contemplation appears as a priority intervention phase, where it is crucial to reduce emotional overload and ambivalence, increase engagement and agency with micro-goals and guided planning, and helping the patient to reframe self-care monitoring from a reactive, anxiety-driven process into a self-regulatory process directed toward action. Interventions aimed at increasing engagement and the sense of agency could facilitate the transition to more active stages. In Contemplation, the focus should not only be on information and awareness but primarily on activation (micro-goals, concrete plans), emotional regulation, and fostering a sense of agency. Supporting emotional regulation can help patient to reduce the overload associated with hyper-focus on bodily signals and to reinterpret symptoms in a less threatening way. Within a motivational interviewing framework, activation can be promoted through strategies such as reflective listening, eliciting change talk, and exploring discrepancies between current behaviors and personally meaningful goals. Rather than pushing for immediate action, the clinician can validate emotional burden and regulate uncertainty, helping the patient feel understood while gently reinforcing perceived competence and readiness.

In Determination, it is crucial to support motivation toward promoting well-being with emotional support and enhancing self-efficacy, so that increasing distress that may characterize this phase does not hinder entry into the action. This may include supportive counseling focused on enhancing self-efficacy, setting realistic expectations, addressing fears related to physical activity (e.g., pain, risk of injury, symptom exacerbation), and reinforcing early positive experiences with adapted physical activity. In Precontemplation, it may be helpful to work on integrating physical activity into already existing self-care routines, avoiding prescriptive approaches, and fostering a reinterpretation of change as a continuity rather than a disruption in the person’s self-care identity. The goal, therefore, is not to “activate” but to realign: to integrate physical activity within the personal meaning of self-care (“how does it connect to what you already do to stay well?”).

## 5 Conclusion

This study highlights that physical activity change in older adults with chronic conditions is a complex process in which motivational stages, self-care behaviors, engagement, and psychological distress form non-linear configurations. These findings should be interpreted as pertaining to early-stage motivational readiness in a clinical prescription context, rather than as evidence of stage dynamics across the full behavioral change continuum, integrating motivational assessment with relevant psychological and emotional indicators for clinical practice. A clinical understanding of these processes can support more personalized, sustainable physical activity interventions, sensitive to the specific vulnerabilities of older adults with chronic conditions.

## Data Availability

The data that support the findings of this study are available from the corresponding author upon reasonable request. The dataset is not publicly available due to privacy and ethical restrictions related to participant confidentiality.
